# PROTOCOLO DIGITAL PARA PROJETO CONCEITUAL E VALIDAÇÃO DE UMA ÓRTESE TORNOZELO-PÉ

**DOI:** 10.1590/1413-785220253301e285432

**Published:** 2025-02-03

**Authors:** Rui Araújo, Patrícia Maria de Moraes Barros Fucs

**Affiliations:** 1 Santa Casa de São Paulo, School of Medical Sciences (FCMSCSP), Orthopedics and Traumatology Department, São Paulo, SP, Brazil

**Keywords:** Orthoses, Foot, Equipment Design, Computer-Aided Design, Órteses, Pé, Desenho de Equipamento, Desenho Assistido por Computador

## Abstract

**Objective:**

This original article aimed to develop a digital protocol for the conceptual design and validation of Ankle-Foot Orthoses (AFO) using 3D mapping technologies.

**Methods:**

A scanned model of the ankle-foot complex of a 12-year-old child with a drop foot was utilized, along with a generic AFO model from a Computer-Aided Design environment. Autodesk Meshmixer and Fusion software were employed for conceptual design and static load analysis.

**Results:**

The static load analysis using the Von Mises failure criterion on the AFO model with ABS material demonstrated structural integrity under critical loading conditions. The digital protocol facilitated the design of a functional and patient-specific AFO orthosis.

**Conclusions:**

The study successfully established a digital workflow for AFO design and validation, showcasing the potential of 3D technologies in creating customized orthoses for lower limb rehabilitation. **Level of Evidence IV; Descriptive Study**.

## Resumo

**Objetivo:**

Este estudo teve como objetivo desenvolver um protocolo digital para o projeto conceitual e validação de Órteses de Tornozelo-Pé (AFO) utilizando tecnologias de mapeamento 3D.

**Métodos:**

Um modelo digital do complexo tornozelo-pé de uma criança de 12 anos com pé caído foi utilizado, juntamente com um modelo genérico de AFO de um ambiente de Projeto Auxiliado por Computador. Os softwares Autodesk Meshmixer e Fusion foram empregados para o projeto conceitual e análise de carga estática.

**Resultados:**

A análise de carga estática utilizando o critério de falha de Von Mises no modelo de AFO com material ABS demonstrou integridade estrutural sob condições críticas de carga. O protocolo digital facilitou o projeto de uma órtese AFO funcional e específica para o paciente.

**Conclusões:**

O estudo estabeleceu com sucesso um fluxo de trabalho digital para o projeto e validação de AFOs, destacando o potencial das tecnologias 3D na criação de órteses personalizadas para reabilitação dos membros inferiores. **Nível de Evidência IV; Estudo Descritivo.**

## INTRODUCTION

The ankle joint is crucial for human support and locomotion, divided into stance and swing phases.^1^ Disorders in this region can lead to difficulties in daily activities and biomechanical compensations, affecting other areas of the body.^2^ Neuropathic injuries can result in deformities, gait alterations, increased energy expenditure, and limitations in daily activities, impacting quality of life. Foot drop syndrome is an example, characterized by deficiency in leg dorsiflexion, leading to abnormal gait and steppage posture.^3^

The treatment of ankle and foot musculoskeletal disorders aims to reduce symptoms, improve mechanics, and restore participation in activities.^4^ Common therapeutic modalities include the use of orthoses, physiotherapy, nerve stimulation, and, in some cases, surgery.^3^ Ankle-Foot Orthoses (AFOs) play a significant role in the treatment of neurological or traumatic injuries, aiding in ankle stabilization, prevention of deformities, and alignment control, contributing to improved gait and balance.^5^ Over decades, researchers have dedicated efforts to the development of innovative AFOs to promote lower limb rehabilitation.

Custom ankle and foot orthoses simplify the manufacturing process by using patient limb scanning to generate a digital model.^6^ Additive Manufacturing (AM) emerges as a promising technology for producing highly customized orthoses, allowing for better fit and enhanced functionality.^7^ Integrating Assistive Technology (AT) with AM aims to develop customized products that combine functionality, attractiveness, and reduced production times, aiming to facilitate the daily lives of individuals with specific needs.^8^ Recent studies highlight the advantages of manufacturing customized orthoses using 3D technologies for patients with deformities.^9^,^10^ Customizing orthoses according to individual patient needs promotes better adherence and contributes to rehabilitation progress. However, the lack of detailed information on the design and materials of orthoses in studies involving AT is a gap in the literature, compromising the validity of results^11^ and support for healthcare professionals dedicated to the development and application of these technologies.^12^

In parallel, the integration of three-dimensional technology-based workflows in orthopedics and prosthetic medicine offers several advantages. One of them is the ability to perform 3D scans in specific regions and transmit the resulting files to other clinics, facilitating the design and manufacture of orthopedic or prosthetic devices in different locations.^13^,^14^ The use of portable devices for 3D scanning expands access to these technologies in various medical facilities.^7^ Additionally, the ease of storage and access to digital files allows for tracking patient progress and consultation of previous designs, facilitating the creation of creative and unique models.^15^ The ability to adjust the alignment of foot and leg segments separately enables more precise modifications during modeling, as well as allowing for objective quantification of alignment, surpassing conventional methods of visual inspection used in orthopedic device manufacturing.^16^ Therefore, this research aims to propose a digital protocol for the conceptual design of an AFO orthosis and its validation through static load analysis using 3D technologies, aiming to fill this gap in scientific study and improve clinical practices.

## MATERIALS E METHODS

### Ankle-foot joint model

**Figure 1:**
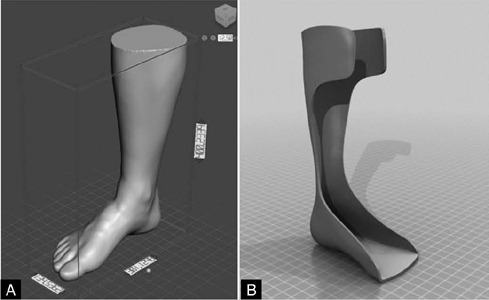
Reference models used: A) Scanned CTP and B) Standard AFO.

For the development of the digital protocol for the conceptual design of the AFO orthosis, a scanned model of the ankle-foot complex (AFC) of a child estimated to be 12 years old, whose disabling condition was drop-foot, was used. This model was provided in partnership with the 3D Technologies Laboratory (LT3D) of the Health Technology Center (NUTES), at the State University of Paraíba (UEPB), and is shown in Figure 1a. The model was obtained after project approval by the Research Ethics Committee in Brazil, with favorable results for its execution (CAAE 21347419.0.0000.5187). The process followed the guidelines of Resolution No. 466/2012, which regulates research involving human subjects, using an informed consent form.

In addition, a generic open-source model of an AFO orthosis developed in a Computer-Aided Design (CAD) environment was also used, obtained from the Ultimaker Thingiverse library. This serves as a reference for the external and internal surfaces of the orthosis, as shown in Figure 1b.

### Conceptual design of the afo model

To develop all the steps related to the conceptual design of the AFO orthosis, from the CTP and AFO models presented earlier, Autodesk Meshmixer 3.5 and Autodesk Fusion software (version 2024) were used. The former was specifically used to perform any mesh correction operations and to convert the model from mesh to a solid model, while the latter was used to carry out the CAD development process of the orthosis itself. It is worth noting that this conversion enables other analyses beyond conceptual design and manufacturing, such as static, modal, impact stress analysis, as well as topological optimization, among other CAE alternatives.^17^

### Validation of the orthesis by static load analysis

For the computational simulation of the new AFO orthosis model, aiming to analyse the interaction between the user’s weight and its reaction on the component, stress and strain analysis using the Von Mises failure criterion is employed. ABS was chosen as the material for this model, as it is a viable option for future manufacturing by AM due to its compatible characteristics for the said application.^18^ The mechanical properties of ABS used in the static analysis of the AFO were defined using standard ASTM sample results.^19^ Autodesk Fusion software was used for this purpose, which offers static analysis capabilities in its CAE (Computer-Aided Engineering) simulation environment. With the obtained results, it is necessary to verify if they meet the convergence criterion, determined based on a safety coefficient and the maximum stress in the model.

For the validation of the designed AFO orthosis model, a static analysis was applied to preliminarily ensure its structural integrity in the face of loads applied in the most critical scenarios of the gait phases, allowing the design to conceive a functional, durable orthosis that meets the patient’s requirements, considering the geometric and material characteristics employed in the model, without expending resources on defective models post-fabrication.^20^ Due to the impossibility of measuring the weight of the patient whose CTP was used in this research, the average value for age and gender of a Brazilian child within the characteristics already presented was used. This allowed for the quantitative establishment of the maximum forces to which the AFO orthosis will be subjected, especially considering the critical cases of stresses applied to the model, which would occur during the midstance phase characterized by the maximum body weight supported on the planted foot in an almost static position and supported on the forefoot.^21^

## RESULTS

### Afo 3d modeling process

**Figure 2:**
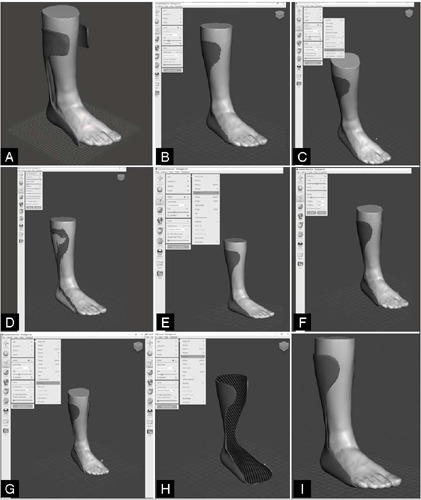
Steps of 3D modeling of the AFO orthosis.

The conceptual design of the AFO orthosis, which involved the 3D modelling process of the customized AFO for the scanned CTP model, consisted of a sequence of steps, as shown in Figure 2.

From the steps shown in Figure 2, it can be observed that step 1 displays the initial model of the standard AFO orthosis, with dimensions adjusted to those of the scanned CTP model, for the preliminary parameterization of the new AFO model. In turn, step 2 shows the active selection tool in Meshmixer, with a highlighted area of the orthosis, which is a possible sketch of the new orthosis model, indicating the beginning of the customization process of the design. The continuation of the selection process, in step 3, shows the consequence of smoothing the selected area in step 2, as refining the mesh of the scanned model makes the sketch too coarse. In step 4, the interface for smoothing edges applied to the selected area is observed, which was used to smooth the transitions and edges of the selection, improving the adaptation of the orthosis to the user and smoothing the layer deposition process during the AM process of the designed AFO. Additionally, step 5 indicates an extrusion operation of the selected area, which properly generates the physical shape of the AFO orthosis for the area outside the boundaries of the scanned model. Step 6 shows that the extrusion was performed on the selected area, as indicated by the overlay of a new geometry over the original area, with a defined thickness. Step 7 consists of essentially separating the previously extruded region from the original CTP model, creating a new model in the software environment that refers only to the AFO orthosis. In step 8, the separated AFO model is analysed individually, so the scanned CTP model is removed from the environment, and to repair the internal surface of the AFO, the external surface is selected and then extruded in the normal direction, to constitute the internal surface of the CAD model of the AFO. Finally, step 9 presents the finalized model after the previous operations. The modified area appears integrated with the rest of the model, indicating the end of the customized design process.

### Static load analysis of the afo model

**Figure 3:**
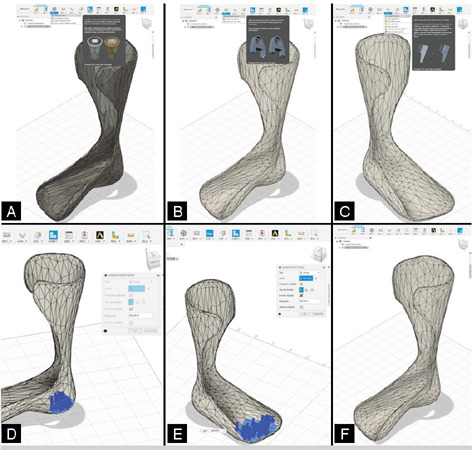
Steps for static load analysis of the AFO orthosis.

Similarly to the AFO orthosis modelling process, its validation involved studying static stresses in the model, assessing critical static loading conditions during the use of the custom orthosis by the patient referenced by the scanned CTP model. This study involves following certain procedures, as depicted in Figure 3.

**Figure 4:**
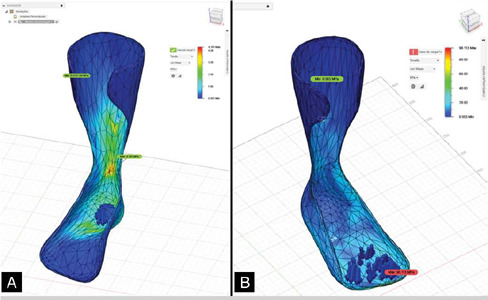
Stress distribution for the AFO model with ABS under loading conditions: A) on the heel and B) on the forefoot.

From the procedures shown in Figure 3, it is highlighted that step 1 refers to preparation and material, in which the AFO model was imported in stl format into Fusion, and the material is defined according to the part in question. In this case, ABS plastic was assigned as the material for the model, determining the physical properties to be considered in the analysis. In step 2, concerning boundary conditions, constraints are applied to the model to simulate how the orthosis will be fixed or where it will be in contact with the skin or other surfaces. In this case, the regions of the AFO in contact with the calf and the longitudinal medial arch. In step 3, regarding the application of static loading, the location and intensity of the loads to be applied are defined, such as gravitational force or some specific force to simulate body weight or pressure exerted during gait. Steps 4 and 5 subdivide the static analysis procedure with two types of statically defined loading. Finally, in step 6, a mesh is created for the model, involving dividing the model into small finite elements to be used in the calculation of the static load analysis. When performing the analysis outlined by the previous steps, the CAE software calculates how the orthosis will respond to the applied loads, and following this simulation, the stress distribution for the AFO model was obtained according to the imposed loading and boundary conditions, as shown in Figure 4.

**Figure 5:**
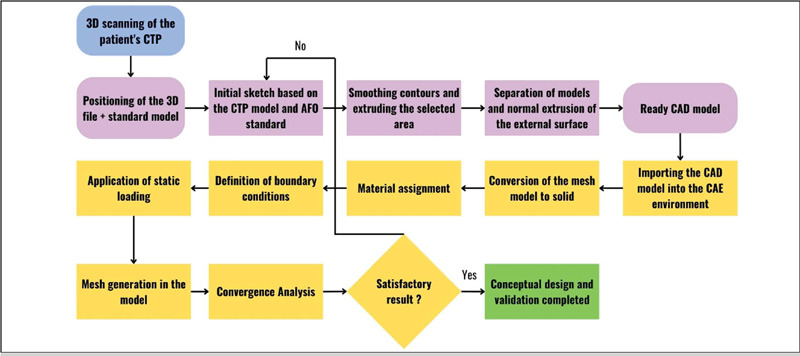
Digital mapping for modelling and validating an AFO model.

After verifying the convergence of the CAD model of the AFO from static loading analysis, it was concluded that the AFO design was validated and could be forwarded for manufacturing. At the end of the design phases addressed in this study, the digital protocol for the design and validation of an AFO presented in Figure 5 is proposed.

## DISCUSSION

The objective of this study was to develop a digital protocol aimed at the conceptual design of an AFO-type orthosis, as well as its validation through static load analysis, with the aid of 3D technologies. Creating a new model of customized AFO from a standardized parameterization model constitutes an approach to the research problem with good scientific acceptability in the O&P field given the design optimization processes, as it is reported that the design of custom or traditional AFOs is typically conducted from the scanned^22^ or measured model using various techniques such as anthropometry,^10^ considering that less experienced professionals may encounter difficulties in parameterizing AFO orthoses without some reference.^23^ The proposed design, being devoid of straps or loops, reduces stress concentrators that may cause component rupture or breakage due to critical cases of static loading or fatigue, as also proposed by Banga et. al (2018).^20^

With regard to validation in CAE environment, other authors have also shown and discussed the loadings used for static analyses,^24^ including considering case studies in children^19^ and applying loading conditions similar to those of the present study. Considering also the stress distribution in the model with the different types of loading, knowing the mechanical properties of the material assigned in this study (ABS plastic, with 38.45 MPa for the yield strength and 43.8 MPa for the ultimate tensile strength), the results were consistent with other researchers who addressed FEA validation for AFO design, especially when considering appropriate boundary conditions. For the present study, the satisfactory result obtained by the CAD model of the AFO is evidenced, as the maximum stress values presented by the static analysis, in Figure 4, show that the assigned material is able to withstand the imposed loading conditions without exceeding the design limit.

## CONCLUSIONS

The study successfully developed a digital protocol for the conceptual design and validation of AFO-type orthoses using 3D technologies. Through static load analysis, the structural integrity of the customized AFO model was ensured, meeting the patient’s requirements. This innovative approach showcases the potential of digital mapping and 3D technologies in designing functional and durable orthoses tailored to individual needs, advancing the field of lower limb rehabilitation.
